# Fabrication of PS/PVDF-HFP Multi-Level Structured Micro/Nano Fiber Membranes by One-Step Electrospinning

**DOI:** 10.3390/membranes13100807

**Published:** 2023-09-22

**Authors:** Yixia Zhao, Zehao Zhang, Yan Zhang, Yuting Huang, Yanfei Chen, Bofei Chen, Weimin Kang, Jingge Ju

**Affiliations:** 1 State Key Laboratory of Separation Membranes and Membrane Processes, National Center for International Joint Research on Separation Membranes, School of Textile Science and Engineering, Tiangong University, Tianjin 300387, China; zhaoyixia@tiangong.cn (Y.Z.); zzh545257873@163.com (Z.Z.); doraemonwaya@126.com (Y.Z.); yuting3480193092@163.com (Y.H.); isyfchen@163.com (Y.C.); chenbofei@outlook.com (B.C.); 2 Shandong Provincial Key Laboratory of Olefin Catalysis and Polymerization, Shandong Chambroad Holding Group Co., Ltd., Economic Development Zone of Boxing County, Binzhou 256500, China

**Keywords:** one-step electrospinning, multi-level structured, micro/nano fiber

## Abstract

Recently, the multi-level interwoven structured micro/nano fiber membranes with coarse and fine overlaps have attracted lots of attention due to their advantages of high surface roughness, high porosity, good mechanical strength, etc., but their simple and direct preparation methods still need to be developed. Herein, the multi-level structured micro/nano fiber membranes were prepared novelly and directly by a one-step electrospinning technique based on the principle of micro-phase separation caused by polymer incompatibility using polystyrene (PS) and polyvinylidene fluoride-hexafluoropropylene copolymer (PVDF-HFP) as raw materials. It was found that different spinning fluid parameters and various spinning process parameters will have a significant impact on its morphology and structures. Under certain conditions (the concentration of spinning solution is 18 wt%, the mass ratio of PS to PVDF-HFP is 1:7, the spinning voltage is 30 kV, and the spinning receiving distance is 18 cm), the PS/PVDF-HFP membrane with optimal multi-level structured micro/nano fiber membranes could be obtained, which present an average pore size of 4.38 ± 0.10 μm, a porosity of 78.9 ± 3.5%, and a water contact angle of 145.84 ± 1.70°. The formation mechanism of micro/nano fiber interwoven structures was proposed through conductivity and viscosity tests. In addition, it was initially used as a separation membrane material in membrane distillation, and its performance was preliminarily explored. This paper provides a theoretical and experimental basis for the research and development of an efficient and feasible method for the preparation of multi-level micro/nano fiber membranes.

## 1. Introduction

In recent years, fiber membranes have attracted widespread attention in various fields [1], such as filtration [2], separation [3], catalysis [4], and biomedicine [5], due to their greater specific surface area, good pore size distribution, and excellent mechanical properties. However, as technology advances and evolves, fiber membranes with a uniform diameter distribution are insufficient for application and are relatively more susceptible, limiting their use in practical applications. As a novel type of fiber membrane material, micro/nano fiber membranes with coarse and fine overlapping multi-stage structures are attracting attention because of their greater specific surface area [6], high porosity [7], high permeability flux [8], and high stability [9]. 

There are many methods [10] for preparing multi-level structured fiber membranes, including the microporous filter membrane method, stretching method [11], template synthesis method [12], electrospinning method [13], chemical vapor deposition method [14], etc. Among them, electrospinning is a commonly used technique for manufacturing nano- or submicron-sized fibers under the condition of applying a strengthened electric field to polymer solutions [15]. Compared with other preparation methods, electrospinning technology is a simple and efficient method to prepare fiber membranes, which has more unique advantages in the preparation of micron and nanoscale fibers, such as a controllable structure, greater specific surface area and surface energy, and lower preparation costs [16,17,18]. Moreover, the process operation is simple and suitable for different types of materials. Due to these advantages, the preparation of electrospun multi-level structured micro/nano fibers has been widely applied in various fields such as materials science [19], mechanical engineering [20], biomedicine [21], etc. In the context of the continuous development of science and technology, electrospinning of multi-level structured micro/nano fiber membranes has become a research hotspot in many fields.

In recent years, researchers have conducted some research on the preparation of multi-level structured micro/nano fiber membranes using electrospinning technology [22]. The most common method is to introduce some additives into the spinning solution to construct a multi-level structure. For example, Qiu et al. [23] used electrospinning technology to prepared the mixed solution of PAN (polyacrylonitrile)/PVP (polyvinylpyrrolidone)/Ni (Ac)_2_ (Ac = acetate) into a multi-level structured fiber membrane material with carbon nanotubes on the surface of the fiber. A et al. [24] researcher used electrospinning technology to prepare a multi-level structured composite membrane composed of a thin active dense layer composed of PVDF-HFP nanofibers and a thick support layer composed of polyethylene terephthalate (PET) microfibers. Multi-level interwoven structured micro/nano fiber membranes can provide good mechanical strength and long-term stability [25]. Our group [26] prepared polyvinylidene fluoride (PVDF) multi-level tree-like nanofibers by adding a certain amount of tetrabutylammonium chloride (TBAC) into the PVDF solution via one-step electrospinning. The introduction of TBAC promotes the formation of dendritic structures, and the fiber membrane has high porosity and high mechanical strength. In addition, some studies combine electrospinning with some other subsequent process modifications to prepare multi-level structured micro/nano fiber membranes. For example, Han et al. [27] prepared fibers based on a blend of a polyetherimide (PEI) solution and a poly (tri butyrate tri light valproate) copolymer (PHBV) solution by thermal degradation combined with electrospinning technology. Due to the thermal degradation of PHBV, the fibers’ membranes were endowed with multi-level structured micro/nano fiber membranes. Zhuge et al. [28] successfully prepared a polyimide (PI)/polyphenylsulfone terephthalamide fiber (PSA) porous micro/nano fiber membrane by electrospinning technology combined with calcination and found that the calcined PI/PSA porous micro/nano fiber membrane had excellent tensile properties and thermal insulation properties. Li et al. [29] used electrospinning PVDF-HFP and spraying a PS and Polydimethylsiloxane (PDMS) mixed solution to prepared a multi-level roughness fiber membrane with a beads-on-string structure. The addition of PDMS during electrospinning can modulate the bead morphology by reducing surface energy as the solvent evaporates, which can effectively improve the antifouling ability and performance stability of fiber membranes. The above studies show that electrospinning is an effective method for preparing micro/nano fibers [30]. The former requires the introduction of additives, which may have a certain impact on the polymer, and the latter also requires an additional process flow during the preparation process, making the preparation process more complex. Therefore, it is particularly important to research and develop an efficient and convenient method for preparing multi-level structured micro/nano fiber membranes.

In this paper, multi-level structured micro/nano fiber membranes were prepared directly by a one-step electrospinning technique using PS and PVDF-HFP as raw materials and based on the principle of micro-phase separation caused by polymer incompatibility. PS/PVDF-HFP nanofiber membranes with coarse and fine overlapping multistage structures exhibit high surface roughness and high porosity. We systematically investigate the effects of key electrospinning parameters including the solution concentration, polymer ratio, voltage, and tip-to-collector distance on the morphology and multi-level structures of the PS/PVDF-HFP nanofiber membranes. Through material characterization, we elucidate the formation mechanism of coarse and fine overlapping structures based on the microphase separation behavior of PS and PVDF-HFP.

PS/PVDF-HFP membranes leverage dual micro/nanoscale fiber morphology to combine advantages from both domains. The nanofibers provide high porosity and a greater surface area, which are beneficial for selectivity, permeability, and antifouling. The microfibers impart mechanical reinforcement. Furthermore, the bicontinuous network interconnects the nano- and micro-domains to create an integrated structure.

Compared to nanofiber-only membranes, we achieve better permeability and durability. Versus microfiber-only membranes, we obtain higher selectivity and a greater surface area. Other blended micro/nanofiber membranes have been reported, but we demonstrate tunable control over the multiscale morphology by adjusting the polymer ratio and electrospinning parameters.

We believe these findings provide new insights into the rational design and fabrication of multi-level structured micro/nano fiber membranes with hierarchical structures for applications such as membrane distillation. It provides a theoretical and experimental basis for the research and development of an efficient and feasible method for the preparation of multi-level structured micro/nano fiber membranes.

## 2. Materials and Methods

### 2.1. Chemicals and Materials

The polystyrene (PS, molecular weight = 2.5 × 10^5^) polymer, polyvinylidene fluoride-hexafluoropropylene copolymer (PVDF-HFP, Mw = 60 w) polymer, and the-N, N-Dimethylformamide (DMF) solvent were, respectively, purchased from Tianjin Guangfu Fine Chemical Research Institute Co., Ltd. (Tianjin, China), Solvay S.A. (Brussels, Belgium), and Tianjin Kermel Chemical Reagent Co., Ltd. (Tianjin, China) in order to fabricate PS/PVDF-HFP nanofiber membranes. The sodium chloride (NaCl) and n-butyl alcohol used during the characterization process were purchased from Shanghai Aladdin Biochemical Technology Co., LTD (Shanghai, China). The water used in this work was distilled water.

### 2.2. Preparation of PS/PVDF-HFP Nanofibers Membranes

**Figure 1:**
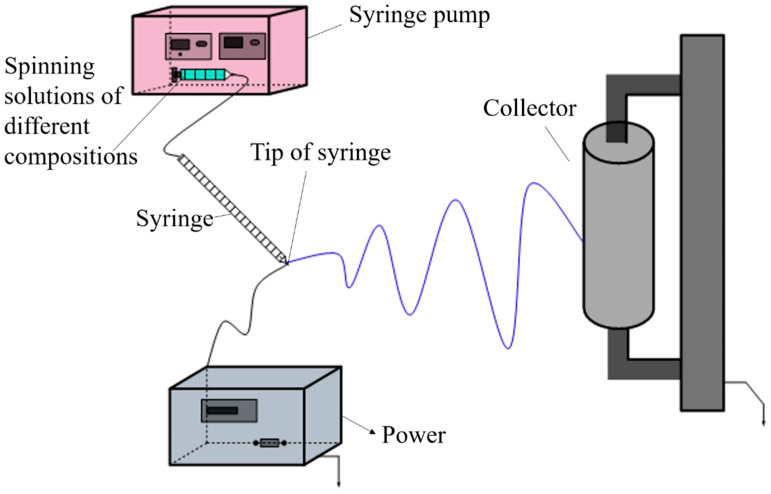
Schematic diagram of electrostatic spinning experimental equipment.

The PS/PVDF-HFP spinning solutions were prepared by simply and directly dissolving mixtures of PS and PVDF (as solute) with different mass ratios (PS: PVDF-HFP = 1:3, 1:5, 1:7, and 1:9) into DMF (as solvent) with various concentrations (14 wt%, 16 wt%, 18 wt%, and 20 wt%). Subsequently, the obtained PS/PVDF-HFP spinning solutions were transferred to the laboratory homemade electrospinning equipment, which was employed with specific electrospinning parameters (voltage of 30 kV, receiving distance of 18 cm, and liquid extrusion rate of 1 mL/h) under an experimental temperature of 25 ± 5 °C and a relative humidity of 40 ± 5%. A schematic diagram of the equipment is shown in Figure 1.

During the electrospinning process, PS/PVDF-HFP micro/nano fibers are continuously deposited and interweaved on the aluminum foil covering the metal spinning roll. The nano- and micro-scale fibers are formed concurrently during spinning through microphase separation and solidify as an interpenetrated network with the two polymer phases intimately mixed. This integrated fabrication method generates a robust membrane structure. In order to be convenient for recording, the electrospun fiber membranes prepared from different ratios of PS/PVDF-HFP spinning solutions (PS: PVDF-HFP = 1:3, 1:5, 1:7, and 1:9) were named as PS/3PH membrane, PS/5PH membrane, PS/7PH membrane, and PS/9PH membrane, respectively. For comparison, the pristine PS membrane and pristine PVDF-HFP membrane were also prepared under the same conditions, which were named as PS membrane and PH membrane, respectively, as shown in Table 1.

In this work, we focused our parametric studies on voltage, collector distance, solution concentration, and polymer ratio, while keeping the flow rate constant. This choice was made to limit the number of variables and establish a baseline process–structure relationship as well as due to the constraints on the adjustable flow rate range to avoid process instabilities like droplet formations.

### 2.3. Characterizations of Membranes

The surface morphology of the fibers was observed using a cold field emission scanning electron microscope (Regulus 8100, Hitachi, Japan), and metal ions were sprayed using an ion sputterer (Blatc SCD005, BAL-TEC, Pfäffikon, Switzerland) for 30 s to enhance electrical conductivity. An image analysis software (Image J 1.52) was used to measure the diameter of 200 randomly selected fibers and to analyze their diameter distribution. The elemental-mapping was characterized by energy disperse spectroscopy (EDS) connected to the above FESEM. A cold-field characteristic X-ray energy spectrometer (Ultim Max 65, Oxford, UK) was used to take surface scan images of the fiber membrane for analyzing its surface elements. A Nicolet iS50 FT-IR spectrometer was used to test the FT-IR spectra of the fiber membranes in order to analyze the functional groups and chemical bonds contained in them. A capillary flow pore size analyzer (Porolux 1000, Prometric Ltd., Belgium, Germany) was used to test the pore size distribution of the fiber membranes. The porosity of the fiber membrane was tested by the weight method. The porosity is calculated using the following formula:

$$\varepsilon =\frac{V_{pore}}{V_{total}}=\frac{m_{2}-m_{1}}{A\times d\times \rho }$$

where *ε* is the porosity of the fiber membrane, %; *m*_1_ and *m*_2_ are the masses of the fiber membrane before and after soaking in n-butanol, g; *A* is the effective membrane area of the fiber membrane to be tested, 9 cm^2^; *d* is the thickness of the fiber membrane before immersion, µm; and *ρ* is the density of n-butanol, 0.81 g·cm^−3^.

To determine porosity, we first precisely weighed the dry membrane samples in petri dishes. We then soaked the membranes in water for 24 h, gently blotted the surface moisture, and weighed the soaked membranes in the same petri dishes.

The strength and elongation at the break of the fiber membrane were tested with a single fiber strength meter (YG005, Bain Instrument Co., Ltd., Wenzhou, China) with a pre-tension of 0.3 N, a gauge of 20 mm, and a tensile speed of 10 mm/min. The breaking strength of the tested fiber membrane was calculated using the following formula:

$$\sigma =\frac{P}{b\times d}$$

where *σ* is the breaking strength of the fiber membrane, MPa; *P* is the breaking strength of the fiber membrane, cN; *b* is the width of the fiber membrane, 0.5 cm; and *d* is the thickness of the fiber membrane, μm.

A fully automatic contact angle measuring instrument (DSA30S, KRUSS, Hamburg, Germany) was used to test the static water contact angle of the fiber membrane using about 5 μL of water drops on the surface of the sample. A true color confocal microscope (CSM700, Zeiss, Oberkochen, Germany) was used to take 3D color images of the surface roughness of the fiber membrane. The DJS-1C conductivity tester and the NDJ-8S rotary viscometer were used to test the conductivity and the viscosity of the spinning solution, respectively.

### 2.4. Direct Contact Membrane Distillation Performance Test

The MD performance of the as-fabricated membranes was evaluated using a lab-scale DCMD system. The laboratory self-assembled DCMD system consisted of a computer, a transparent mold, a peristaltic pump, an electronic balance, a conductivity meter, a water bath, and a thermostatic water tank, and the effective membrane test area was 4 cm^2^. The DCMD experiments were performed with a 35 g·L^−1^ NaCl solution as the feed solution, and peristaltic pumps were used to circulate both the feed solution and the permeate solution whose flow rates were both set to 0.55 L·min^−1^. A thermostatically heated magnetic stirrer was used. The feed solution was kept at 60 °C and the permeate was kept at 20 °C using a constant temperature water tank. The permeate flux (J, L·m^−2^-h^−1^) and the salt rejection (R, %) were recorded by computer and calculated using the following equation from the change in mass and conductivity of the permeate, respectively.

$$J=\frac{\Delta M}{\Delta T\times S}$$

where Δ*M* is the weight gain of the permeate, kg; Δ*T* is the operation time of the DCMD system, h; and *S* is the effective test area of the fiber membrane to be tested, 4 cm^2^.

$$R=\frac{EC_{feed}-EC_{distillate}}{EC_{feed}}\times 100\%$$

where *EC_distillate_* and *EC_feed_* are the conductivity of the permeate and feed solutions, %, respectively.

## 3. Results and Discussion

### 3.1. Effect on Fiber Membrane Morphology

**Figure 2:**
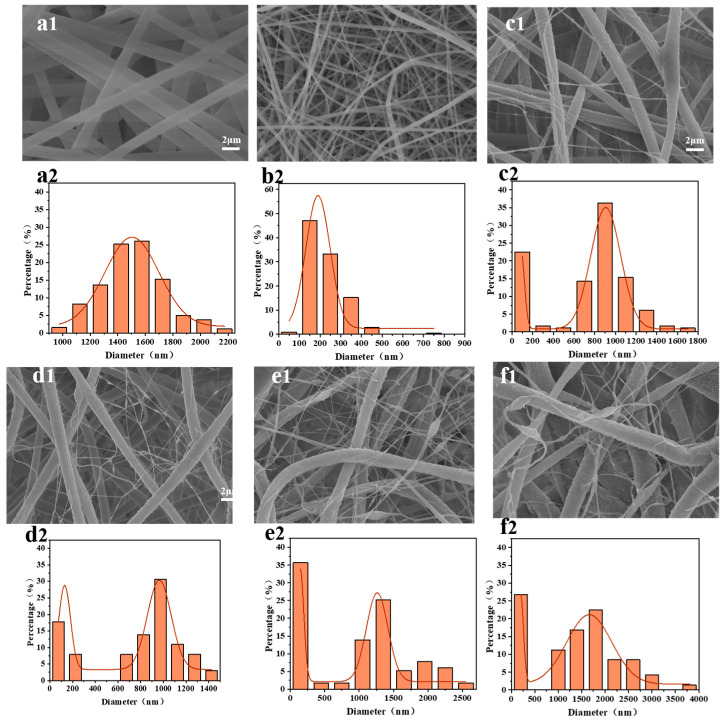
Scanning electron microscopy and fiber diameter distributions of fiber membranes prepared with different spinning solution ratios at a spinning solution concentration of 18%: (**a**) PS, (**b**) PH, (**c**) PS/3PH, (**d**) PS/5PH, (**e**) PS/7PH, and (**f**) PS/9PH, for each spinning solution. (1) Scanning electron microscope image of the fiber membranes, (2) Fiber diameter distributions.

The surface morphology and diameter distribution of the PS/PVDF-HFP fiber membranes with different spinning solution ratios in this study are shown in Figure 2. It can be seen that the fibers of both PS membranes (Figure 2a) and PH membranes (Figure 2a) are continuous and uniform and present narrow diameter distributions with average diameters of approximately 1500 ± 200 nm and 200 ± 100 nm, respectively. When the spinning solutions of PS and PH were blended, the morphologies of the fiber membranes showed significant changes which presented the phenomenon of interweaving between microfibers (500–3000 nm) and nanofibers (100–200 nm). The influence of the spinning solution ratio on the spinning process is mainly reflected in the properties of the polymer itself and the viscosity fluctuations of the spinning jet. As the proportion of PVDF-HFP in the spinning solution increases, the proportion of nanofibers in the fiber membrane gradually increases, and the diameter range of microscale fibers shifts to the range of 1000–2000 nm. Specifically, it can be seen that when the mass ratio of PS to PVDF-HFP is 7:1, a large number of nanofibers appear in the fiber membrane, and the average diameter of microfibers increases to 1200 ± 300 nm. However, when the mass ratio of PVDF-HFP to PS increases to 9:1, the overall diameter of the fiber membrane increases, and fiber adhesion occurs. This is because the viscosity of the spinning solution is too high and the fibers are not fully stretched, leading to the incomplete evaporation of the solvent.

**Figure 3:**
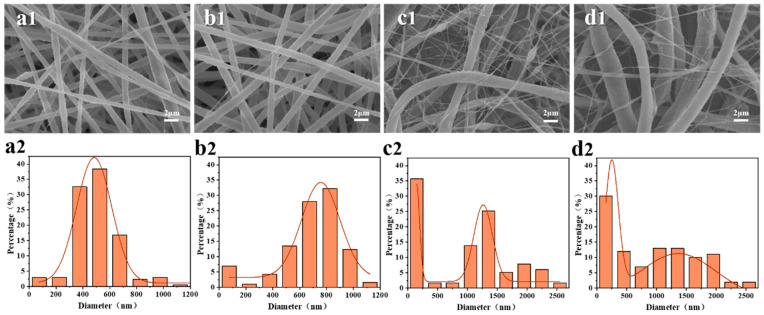
(1) Scanning electron micrographs and (2) fiber diameter distributions of PS/7PH membranes fabricated at different spinning solution concentrations at an acceptance distance of 18 cm and a voltage of 30 kV: (**a**) 14 wt%, (**b**) 16 wt%, (**c**) 18 wt%, and (**d**) 20 wt%.

The formation of the PS/PVDF-HFP nanofiber membrane is related to the concentration of the spinning solution, which affects its rheological properties. A low concentration can easily form beaded fibers, while a high concentration can lead to an increase in fiber diameter. The surface morphology and diameter distribution of membranes prepared under different spinning solution concentrations are shown in Figure 3. It can be seen in Figure 3a that when the concentration is 14 wt%, the fibers in membrane present a uniform diameter between 300–700 mm, but a small number of beads are formed. When the concentration increases to 16 wt%, the number of beads decreases significantly, the fiber diameter increases overall, and it is concentrated between 500–1000 nm. With the increase of the spinning solution concentration to 18 wt%, a large number of nanofibers appear in the membrane, and the fiber diameter is distributed in a two-stage pattern (nanofibers are distributed between 100–200 nm, and microscale fibers are distributed between 900–1500 nm). By further increasing the concentration to 20 wt%, all of the fibers in the membrane show an increase in diameter, accompanied by a decrease of nanofibers and the adherence of some fibers. Overall, the fiber diameter in the PS/PVDF-HFP membrane prepared by the one-step electrospinning method reached the micro/nano stage, forming a micro/nano fiber interweaving structure. The higher the concentration of the spinning solution, the more obvious the micro/nano fiber interweaving structure. This is because of the incompatibility between the two polymers, which results in the occurrence of unstable turbulent eddy currents at the nozzle after the formation of Taylor cones during the spinning process [31]. When the spinning solution concentration is low, the viscosity and number of polymer entanglements are relatively low, which results in the formation of beaded fibers. As the spinning solution concentration increases, the viscosity and number of polymer entanglements also increase, which favors the formation of continuous fibers with an increased diameter [32]. The surface of fibers obtained at different concentrations of the spinning solution exhibits a secondary structure similar to grooves and folds. The appearance of this structure is due to the small diameter and greater specific surface area of the fibers promoting solvent evaporation during the spinning process, resulting in a decrease in the surface temperature of the fibers. Water molecules in the atmosphere, as poor solvents for PS and PVDF-HFP, will induce phase separation and form a rough surface.

**Figure 4:**
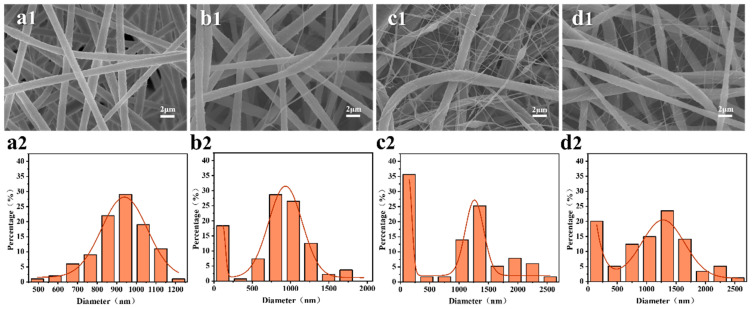
(1) Scanning electron micrographs and (2) fiber diameter distributions of PS/7PH membranes fabricated at different spinning voltages with an acceptance distance of 18 cm and a spinning solution concentration of 18 wt%: (**a**) 20 kV, (**b**) 25 kV, (**c**) 30 kV, and (**d**) 35 kV.

In addition to the concentration of the spinning solution, the applied spinning voltage determines the smooth progress of the spinning process and the stability of the spinning jet, thereby affecting the morphology of the fiber membrane. Figure 4 shows the surface morphology and diameter distribution of the PS/7PH membrane prepared at different spinning voltages. It can be seen that when the spinning voltage is 20 kV, the fiber diameter distribution is uniform, which is concentrated at 800–1000 nm. When the spinning voltage was increased to 25 kV, a small number of nanoscale fibers began to appear in the fiber membrane. When the spinning voltage continued to increase to 30 kV, the nanoscale fibers in the fiber membrane increased significantly, and the diameter range of the micron scale fibers gradually moved to the range of 1000–1500 nm. When the spinning voltage continued to increase to 35 kV, the diameter of the micro scale fibers and nanoscale fibers in the fiber membrane increased to 1000–1800 nm and 100–500 nm, respectively. It can be concluded that the relatively high spinning voltage is conducive to the formation of multi-level micro/nano structures, which is because the increase of the spinning voltage increases the instability of the spinning process.

**Figure 5:**
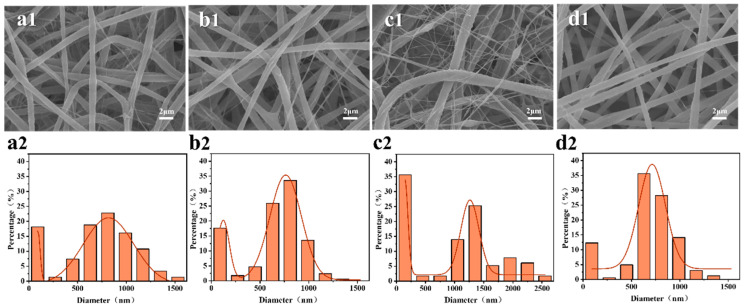
(1) SEM and (2) fiber diameter distributions of PS/7PH membranes fabricated at different spinning acceptance distances at a voltage of 30 kV and a spinning solution concentration of 18 wt%: (**a**) 12 cm, (**b**) 15 cm, (**c**) 18 cm, and (**d**) 21 cm.

Figure 5 shows the surface morphology and diameter distribution of the membranes with different reception distances. It can be observed that when the electrospinning receiving distance is 12 cm, there are relatively few nanofibers and little adhesion between the fibers. This is because the electrospinning receiving distance is too short, the fiber is not fully stretched, and the solvent has not completely evaporated. When the electrospinning receiving distance is increased to 15 cm, the solvent evaporates completely, and the fiber is fully stretched, resulting in a decrease in the diameter of the micron-scale fibers. When the electrospinning receiving distance is further increased to 18 cm, the number of nanofibers in the fiber membrane increases significantly, and the diameter range of the micron-scale fibers gradually shifts towards the range of 1000–1500 nm. When the electrospinning receiving distance continues to increase to 21 cm, the diameter of the micron-scale fibers in the fiber membrane decreases and shifts towards the range of 600–1000 nm, and the number of nanofibers decreases significantly. This is because the electrospinning receiving distance is too large, which weakens the electric field strength and thus decreases the instability of the electrospinning. It could be concluded that both too-close and too-far electrospinning receiving distances are not conducive to the formation of multi-level micro/nano structures. After a discussion, it was found that the multi-level structured micro/nano fiber membranes can be effectively prepared when the spinning solution concentration is 18 wt%, the spinning voltage is 30 kV, the receiving distance is 18 cm, and the mass ratio of PVDF-HFP to PS is 7:1.

**Figure 6:**
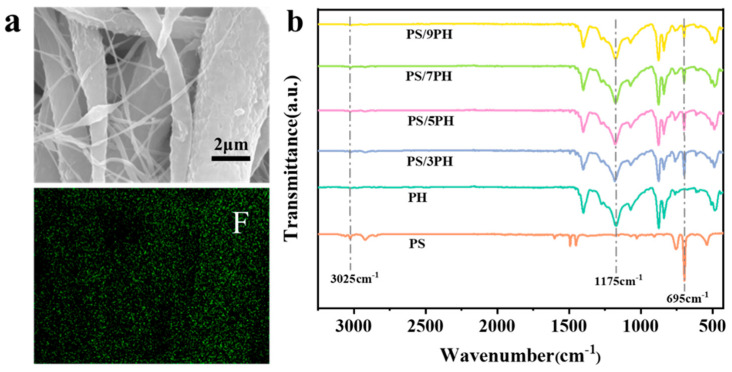
(**a**) Element mapping analysis of PS/7PH membrane; (**b**) Infrared spectra of fiber membranes prepared with different spinning solution ratios.

In order to verify the distribution of the fibers in the membrane, the chemical composition of the sample was characterized using X-ray characteristic spectroscopy and Fourier transform infrared spectroscopy. The element mapping analysis of the PS/7PH membrane in Figure 6a shows the presence of an F element that originated from PVDF-HFP in both the micron and nanofibers, indicating that PVDF-HFP and PS are uniformly distributed throughout the nanofiber membrane in a co-blended form. The FTIR spectra of the fiber membranes prepared with different spinning solution ratios are shown in Figure 6b. It can be seen that there are characteristic peaks that appear at 1175 cm^−1^, 2870–3100 cm^−1^, 840 cm^−1^, and 877 cm^−1^, corresponding to the stretching vibrations of the CF_2_ group [33], CH_2_ group [34], β phase, and α phase of PVDF in PVDF-HFP [35], respectively, which indicates that PVDF-HFP is successfully introduced into the nanofiber membrane. In addition, the characteristic peaks that appeared at 3025 cm^−1^ and 695 cm^−1^, respectively, correspond to a symmetrical stretching vibration of the C-H groups in aromatic rings [36] and a bending peak of the C-H groups in mono-substituted aromatics [37]. The gradual weakening of the C-H bending peak with decreasing PS content suggests that PS is also successfully introduced into the nanofiber membranes.

### 3.2. Properties of PS/PVDF-HFP Nanofiber Membrane

**Figure 7:**
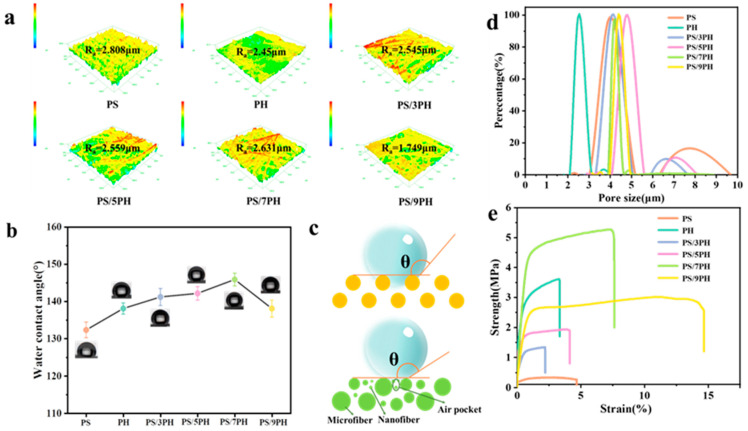
Fiber membranes prepared with different spinning solution ratios: (**a**) Surface three-dimensional confocal microscope images and (**b**) static water contact angle; (**c**) Schematic diagram of water droplets on different membrane surfaces; (**d**) Pore size distribution diagram and (**e**) stress–strain curve diagram.

The surface wettability of the membrane was evaluated using static contact angle, as shown in Figure 7, where the water contact angle of the PS membrane is the smallest (132.30 ± 2.10°). The water contact angles of the PH membrane, PH/3PH membrane, PS/5PH membrane, and PS/7PH membrane are 138.05 ± 1.50°, 141.25 ± 2.30°, 142.11 ± 1.80°, and 145.84 ± 1.70°, respectively. According to the Wenzel/Cassie model [38,39,40], rough surfaces can further enhance the hydrophobicity of hydrophobic fiber membranes. Figure 7a shows the surface three-dimensional confocal microscope images of the fiber membranes prepared with different spinning solution ratios. The Ra values of the PH membranes, PS/3PH membranes, PS/5PH membranes, and PS/7PH membranes are 2.45 μm ± 0.02, 2.545 μm ± 0.02, 2.559 μm ± 0.02, and 2.631 μm ± 0.02, respectively. This indicates that multi-level micro/nano structures can improve the surface roughness of fiber membranes. However, due to the reduction in nanofibers and fiber adhesion, the roughness of the PS/9PH membrane decreases (Ra = 1.749 μm). The trend of the water contact angle is similar to the roughness results. This is because the multi-level micro/nano structure composed of micrometer scale fibers and nanofibers increases surface roughness, leading to the formation of air pockets, and the air pockets increase the contact area of the air–water interface, thereby increasing the water contact angle. Overall, the PS/7PH membrane exhibits the most excellent hydrophobic performance due to its multi-level micro/nano structure and high roughness, and the roughness of the PS/9PH membrane decreases, resulting in a decrease in its water contact angle (138.04 ± 2.30°) due to the reduction in nanofibers and fiber adhesion.

The pore structure of the membrane was characterized by pore size distribution and porosity, and the results are listed in Table 2 and Figure 7d. In terms of pore size distribution, it can be seen that the PH membrane presents the smallest average pore size of 2.77 ± 0.10 μm with a narrow pore size distribution (ranging from 2.1 to 4.0 μm). In comparison, the average pore size of the PS membrane is 4.52 ± 0.20 μm with a wider pore size distribution (ranging from 3.8–6.3 μm). When PVDF-HFP blends with PS, the average pore size of the PS/3PH composite membrane increases from 4.54 ± 0.10 μm. Moreover, as the mass ratio of PVDF-HFP to PS increases to 5:1, the average pore size of the fiber membrane increases to 5.74 ± 0.20 μm. This is due to the gradual increase of micron scale fiber diameter, and a larger diameter reduces the bulk density of the fiber membrane, thus reducing the average pore size. When the mass ratio of PVDF-HFP to PS increases to 7:1, the average pore size of the former decreases to 4.38 ± 0.10 μm compared to the PS/5PH membrane. This is because there are more nanofibers in the PS/7PH membrane, which reduces the pore size. However, when the mass ratio of PVDF-HFP to PS continues to increase to 9:1, the average pore size slightly increases to 4.64 ± 0.20 μm. This is because the nanoscale fibers in the fiber membrane decrease, while the micron scale fiber diameter increases slightly, making the pore size increase.

In terms of the porosity of these membranes, in Table 2, it can be seen that the pure PH membrane has the highest porosity (81.9 ± 2.1%), while pure PS membrane has the lowest porosity (74.1 ± 3.2%). In comparison, the porosity of the PVDF-HFP/PS composite membrane is in the middle. Specifically, when the mass ratio of PVDF-HFP to PS increases from 3:1 to 7:1, the porosity of the fiber membrane increases from 76.4 ± 3.1% to 78.9 ± 3.5%, due to an increase in the proportion of nanoscale fibers and the interweaving of microscale and nanoscale fibers, which reduces the stacking density. However, when the mass ratio of PVDF-HFP to PS continues to increase to 9:1, the porosity of the fiber membrane decreases and is lower than that of the pure PS membrane due to a decrease in the nanofiber proportion and a slight increase in the micrometer scale fiber diameter in the fiber membrane.

In addition, the mechanical properties of fiber membranes affect their long-term durability, which is not only determined by the properties of polymers, but also affected by the membrane structure. The stress–strain curves of fiber membranes prepared with different spinning solution ratios are shown in Figure 7e where it can be seen that when the mass ratio of PVDF-HFP to PS increases from 3:1 to 7:1, the proportion of PVDF-HFP increases, and the fracture strength and elongation at the break of the fiber membrane also increase. This is because the multi-level micro/nano structures can improve the mechanical properties of the membrane [26,40]. Namely, microscale fibers provide skeletal support, nanoscale fibers provide attachment support, and nanoscale fibers interact strongly with micron-scale fibers through entanglement and bonding points to improve tensile properties. When the mass ratio of PVDF-HFP to PS continues to increase to 9:1, the elongation at the break of the fiber membrane continues to increase and the fracture strength decreases due to the reduction in the nanofiber proportion in the entire membrane.

### 3.3. Study of Multi-Level Structured Forming Mechanism

**Figure 8:**
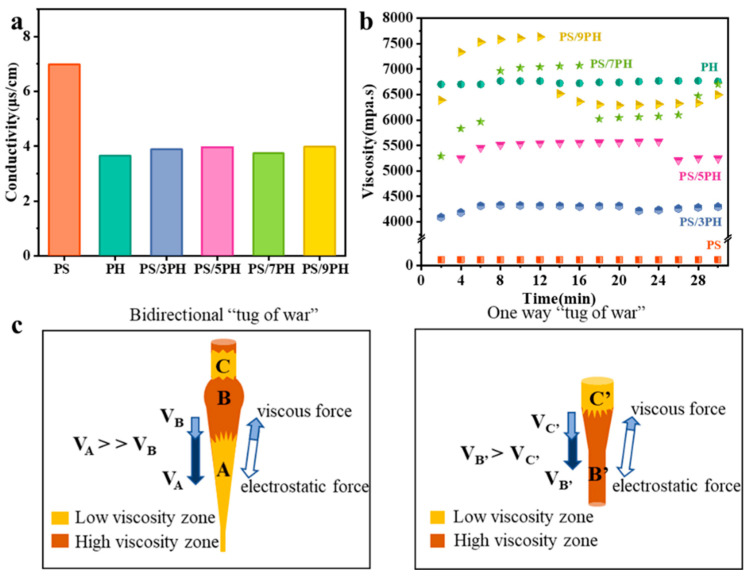
Different ratios of spinning solutions’ (**a**) conductivity and (**b**) viscosity; (**c**) Schematic diagram of the formation mechanism of multi-level micro/nano fiber interweaving structures.

The formation mechanism of micro/nano fibers in the PS/PVDF-HFP nanofiber membranes was investigated through conductivity and viscosity tests on the electrospinning solution. As shown in Figure 8a, the conductivity test results of the different ratios of the electrospinning solutions were obtained. The conductivity of the PS electrospinning solution is 6.98 μS·cm^−1^, and the conductivity of the PVDF-HFP electrospinning solution is 3.65 μS·cm^−1^. When the mass ratio of PVDF-HFP to PS in the blended electrospinning solution increases from 3:1 to 9:1, the conductivity values of the electrospinning solutions are 3.88 μS·cm^−1^, 3.97 μS·cm^−1^, 3.74 μS·cm^−1^, and 3.98 μS·cm^−1^, respectively. These results indicate that the conductivity of the electrospinning solutions is not significantly influenced by the mixture ratio. Figure 8b shows the viscosity test results of the different ratios of the electrospinning solutions. The viscosity of the PS electrospinning solution is approximately 115 mPa·s, while the viscosity of the PVDF-HFP electrospinning solution is approximately 6750 mPa·s. When the mass ratio of PVDF-HFP to PS in the co-blended spinning solution increases from 3:1 to 9:1, significant viscosity fluctuations occur, and the larger the mass ratio of PVDF-HFP in the spinning solution, the greater the viscosity fluctuations.

As for the PS/7PH membrane, the viscosity of the co-blended spinning solution fluctuates roughly within two ranges, 5290–6100 mPa·s and 6700–7071 mPa·s, which are called the low-viscosity and high-viscosity regions, respectively. This is caused by a micro-phase separation phenomenon resulting from the incompatibility between PS and PVDF-HFP. As shown in Figure 8, when the low-viscosity region A is in a stretch-thinning state, the spinning jet is stretched and refined under the action of electrostatic forces. Meanwhile, it also experiences strong resistance from the higher-viscosity region B, with V_A_ significantly larger than V_B_. This process is similar to bidirectional tugging, resulting in the refinement of region A to the nanofibers. When the high-viscosity region B’ is in a stretch-thinning state, the spinning jet is refined by electrostatic forces and smaller viscous forces from the low-viscosity region C’, resulting in a small velocity difference between V_B’_ and V_C’_. This process is similar to unidirectional tugging, making it difficult to refine the fibers. As a result, region B’ forms micron-scale fibers [40,41]. Consequently, electrospinning parameters including voltage and collector distance also play a critical role by dictating the stretching and thinning forces. Furthermore, factors such as solution concentration and flow rate affect jet stability.

**Figure 9:**
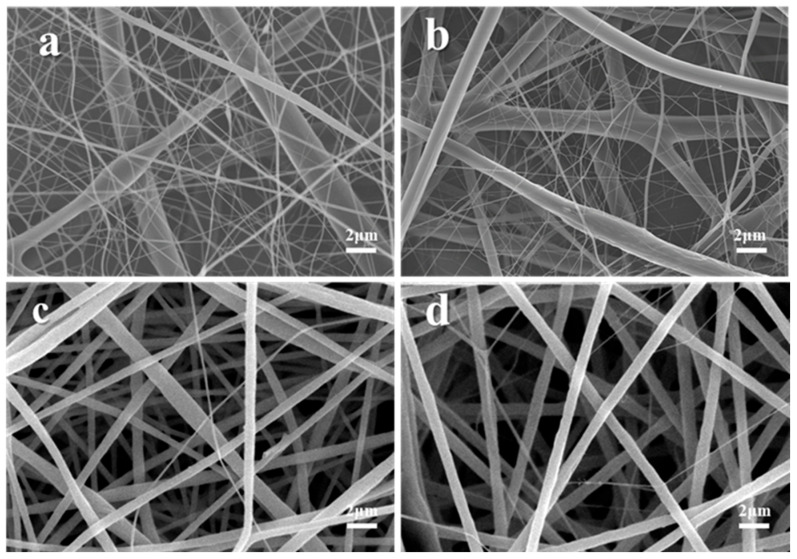
PS/PVP fiber membranes prepared with different voltages at a spinning solution concentration of 12.5 wt%; (**a**) 22kV; (**b**) 30 kV; PAN/PU fiber membranes prepared with different spinning solution concentrations of (**c**) 16 wt% and (**d**) 18 wt%.

In order to verify the generality of the method, relevant experiments were carried out with other incompatible polymers such as polyacrylonitrile/polyurethane (PAN/TPU) and polystyrene/polyvinylpyrrolidone (PS/PVP). As shown in Figure 9, it is obvious that they can also be spun into micro/nano fiber membranes with multi-level structures via a one-step electrospinning technology by combining the regulation of spinning parameters such as the concentration and ratio of the spinning solution, voltage, extrusion rate, and receiving distance in the electrospinning process, which contribute to a more comprehensive understanding of the nature of the mechanism and also further corroborate the universality of the formation mechanism.

Eventually, a structure of interwoven nanofibers and microfibers is formed in the membrane, which presents better mechanical properties, more hydrophobic properties, and better pore structure than pure nanofibers and pure micron fibers. The modular tunability of the membrane morphology could enable optimization for different performance requirements. It would be valuable to explore our optimized membrane in some of these other application areas.

### 3.4. Direct Contact Membrane Distillation Performance Analysis

**Figure 10:**
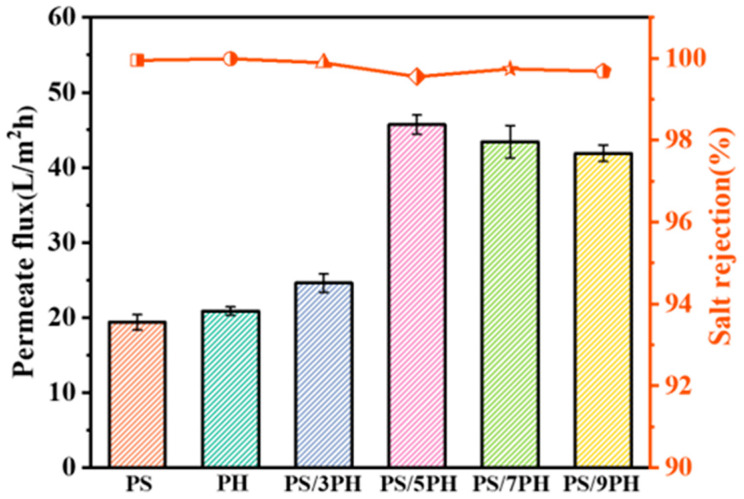
DCMD performance of fiber membranes produced with different spinning solution ratios.

In order to verify the feasibility of multi-level micro/nano fiber applications, its performance as a separation membrane applied in membrane distillation for desalination was initially investigated. The 3.5 wt% NaCl solution was used as the feed solution to test the DCMD performance of the fiber membranes prepared with different spinning solution proportions. The temperatures of the feed solution and permeate solution were 60 °C and 20 °C, respectively. After stabilizing for 1 h, the DCMD performance test results (1 h) of the fiber membrane were recorded, as shown in Figure 10. It can be seen that the permeation flux of the PS membrane and PH membrane is not significantly different, with values of 19.38 ± 1.04 L·m^−2^·h^−1^ and 20.87 ± 0.57 L·m^−2^·h^−1^, respectively, and the salt rejections are 99.95% and 99.99%, respectively. After blending PS and PVDF-HFP for spinning, the DCMD permeate flux of the PS/3PH and PS/5PH membranes are 24.59 ± 1.23 L·m^−2^·h^−1^ and 45.71 ± 1.29 L·m^−2^·h^−1^, with a salt rejection of 99.89% and 99.55%. Compared to the pristine PS microfiber membrane and pristine PVDF-HFP nanofiber membrane, the membrane with a multi-level micro/nano fiber interleaving structure can reduce the mass transfer resistance and the increased heat transfer resistance due to its high porosity, good average pore size, and excellent strength, which in turn leads to an increase in the permeate flux. However, the large pore size makes it easy for saline water to pass through the fiber membrane, resulting in a lower salt rejection. When continuing to increase the proportion of PVDF-HFP in the spinning solution as PS/7PH and PS/9PH, the permeate fluxes could reach 43.41 ± 2.17 L·m^−2^·h^−1^ and 41.89 ± 1.09 L·m^−2^·h^−1^, with a salt rejection of 99.74% and 99.68%, respectively. This is because with a more significant multi-level micro/nano structure, the porosity of the PS/7PH membrane slightly increases and the average pore size decreases significantly, thus increasing the mass transfer resistance and resulting in a slight decrease in the permeate flux. Moreover, the average pore size and porosity of the PS/9PH membrane are significantly decreased, resulting in a further increase in the mass transfer resistance and a significant reduction in the permeate flux. In addition, the average pore size and porosity of the PS/9PH membranes are significantly reduced, leading to a further increase in the mass transfer resistance and a significant decrease in the permeate flux. The above preliminary studies have confirmed the feasibility and advancement of a membrane with a multi-level micro/nano fiber interleaving structure in membrane distillation applications. However, this study is obviously not comprehensive enough. A consideration of the distillation performance must also examine the contamination resistance of the diaphragm. The next phase of this project should be directed towards a series of robust BSA contamination tests under an extended set of membrane compositions and conditions. Comparing fouling profiles will generate important insights into the relationships between material properties, processing factors, and long-term membrane stability. Subsequent research will continue to systematically investigate the performance (such as long cycle time, anti-fouling, etc.) and theory (heat transfer, mass transfer, etc.) of multistage fiber membranes in membrane distillations.

## 4. Conclusions

In conclusion, based on the principle of microphase separation caused by polymer incompatibility, PS/PVDF-HFP nanofiber membranes were prepared through the one-step electrospinning technology.

This experiment found that when the concentration of the spinning solution was 18 wt%, the mass ratio of PS to PVDF-HFP was 1:7, the spinning voltage was 30 kV, and the spinning receiving distance was 18 cm. The optimal multi-level micro/nano fiber interweaving structure of the PS/PVDF-HFP membrane was obtained, which presents an average pore size of 4.38 ± 0.10 μm, a porosity of 78.9 ± 3.5%, and a water contact angle of 145.84 ± 1.70°.

The forming mechanism of this multi-level micro/nano fiber interweaving structure might be explained by the incompatibility between PS and PVDF-HFP causing microphase separation, which leads to the viscosity fluctuation of the spinning jet during electrospinning. Under the combined action of electrostatic force and viscous force, the high-viscosity region and low-viscosity region form the interwoven structure of the micro/nano fiber. Its application as a separation membrane in the field of membrane distillation was preliminarily investigated, which produced a permeation flux of 43.41 ± 2.17 L·m^−2^·h^−1^ and a salt rejection rate of 99.74%.

This study provides a novel idea for the research and design of multistage micro/nano fiber interwoven structures prepared by other polymer blends.

## Figures and Tables

**Table 1:** Experimental scheme.

Solution Concentration (%)	Solution Ratio PS: PVDF-HFP	Voltage (kV)	Reception (cm)
	1:7	30	18
16	1:7	30	18
18	1:7	30	18
20	1:7	30	18
18	1:3	30	18
18	1:5	30	18
18	1:9	30	18
18	1:7	20	18
18	1:7	25	18
18	1:7	35	18
18	1:7	30	12
18	1:7	30	15
18	1:7	30	21

**Table 2:** Membrane parameters.

Membrane	PS	PH	PS/3PH	PS/5PH	PS/7PH	PS/9PH
Thickness (μm)	250 ± 20	250 ± 20	250 ± 20	250 ± 20	250 ± 20	250 ± 20
Mean pore size (μm)	4.52 ± 0.2	2.77 ± 0.10	4.54 ± 0.10	5.74 ± 0.20	4.38 ± 0.10	4.64 ± 0.20
Maximum pore size (μm)	6.32 ± 0.2	4.00 ± 0.10	5.92 ± 0.10	8.12 ± 0.20	5.26 ± 0.10	5.34 ± 0.20
Porosity (%)	74.1 ± 3.2	81.9 ± 2.1	76.4 ± 3.1	77.8 ± 3.5	78.9 ± 3.5	72.8 ± 2.3

## Data Availability

The data that support the findings of this study are available from the corresponding author upon reasonable request.
